# Optimization of polyhydroxyalkanoate production in *Halomonas* sp. YLGW01 using mixed volatile fatty acids: a study on mixture analysis and fed-batch strategy

**DOI:** 10.1186/s12934-023-02188-x

**Published:** 2023-09-04

**Authors:** Yerin Park, Jong-Min Jeon, Jea-Kyung Park, Yung-Hun Yang, Shin Sik Choi, Jeong-Jun Yoon

**Affiliations:** 1 Green & Sustainable Materials R&D Department, Korea Institute of Industrial Technology (KITECH), Cheonan-si, Chungnam 31056 Republic of Korea; 2 Department of Food and Nutrition, Myongji University, Yongin-si, 17058 Republic of Korea; 3 Department of Biological Engineering, Konkuk University, Seoul, 05029 Republic of Korea

**Keywords:** Polyhydroxyalkanoate, *Halomonas* sp., Volatile fatty acids

## Abstract

Polyhydroxyalkanoate (PHA) is one of the most promising materials for replacing petroleum-based plastics, and it can be produced from various renewable biomass sources. In this study, PHA production was conducted using *Halomonas* sp. YLGW01 utilizing mixed volatile fatty acids (VFAs) as carbon sources. The ratio and concentration of carbon and nitrogen sources were optimized through mixture analysis and organic nitrogen source screening, respectively. It was found that the highest cell dry weight (CDW) of 3.15 g/L and PHA production of 1.63 g/L were achieved when the ratio of acetate to lactate in the mixed VFAs was 0.45:0.55. Furthermore, supplementation of organic nitrogen sources such as soytone resulted in a ninefold increase in CDW (reaching 2.32 g/L) and a 22-fold increase in PHA production (reaching 1.60 g/L) compared to using inorganic nitrogen sources. Subsequently, DO-stat, VFAs consumption rate stat, and pH–stat fed-batch methods were applied to investigate and evaluate PHA productivity. The results showed that when pH–stat-based VFAs feeding was employed, a CDW of 7 g/L and PHA production of 5.1 g/L were achieved within 68 h, with a PHA content of 73%. Overall, the pH–stat fed-batch strategy proved to be effective in enhancing PHA production by *Halomonas* sp. YLGW01 utilizing VFAs.

## Introduction

Plastic is an essential material in a variety of industries in modern society. However, petroleum-based plastic takes an extensively long time to naturally decompose, leading to environmental pollution, destruction of marine ecosystems, and greenhouse gas emissions [1, 2]. In particular, as part of a global effort towards climate change mitigation and achieving carbon neutrality, biodegradable plastics are being actively explored as a substitute for petroleum-based plastics.

Polyhydroxyalkanoate (PHA) is a biopolymer biosynthesized by microorganisms and is considered an alternative to traditional plastic due to its comparable physical properties. It can be produced from a wide range of biomass sources, and the composition of monomers such as 3-hydroxybutyrate (3HB), 4-hydroxybutyrate (4HB), 3-hydroxyvalerate (3HV), 3-hydroxyhexanoate (3HHx), and others allows for the creation of various copolymers [3–5]. In addition, PHA exhibits relatively superior biodegradability compared to other bioplastics such as polylactic acid (PLA), polybutylene adipate terephthalate (PBAT), and polybutylene succinate (PBS) [6–8]. Despite the aforementioned advantages, PHA currently holds a modest 3.9% share in the global bioplastics market as of 2022, primarily due to its high production cost [9]. To address this challenge, extensive research is underway to reduce PHA production costs by using diverse biomass sources. Among the various renewable biomass sources, organic acids are recognized as one of the most abundant carbon sources. Consequently, several studies have reported the production of PHA using strains such as *Cupriavidus necator*, *Escherichia coli*, *Halomonas* species, *Cobetia* species and *Bacillus* species by harnessing the potential of organic acids as feedstocks [10–17]. In particular, in case of the food waste, short-chain volatile fatty acids (VFAs) such as acetate, lactate, propionate, and butyrate are present in a mixed state [18, 19]. Through research on PHA production using these mixed VFAs, it has been reported that *C. necator* H16 strain can produce 93.5 g/L of PHB, and other strains such as *Salinivibrio* spp. TGB19 have been reported to produce 53.23 g/L of PHB [20, 21]. In an attempt to lower the production cost, PHA production from VFAs using *Halomonas* strains was reported [13]. *Halomonas* strains are capable of growth even under high salinity conditions, where the survival of other microorganisms is exceedingly difficult. This characteristic eliminates the need for the sterilization of culture equipment, contributing to cost reduction in PHA production. In a previous study, *Halomonas* sp. YLGW01 were shown to achieve PHA contents of up to 95% when utilizing glucose or fructose as carbon sources [22]. Additionally, it was demonstrated that PHA production is feasible using key VFAs such as acetate, lactate, and propionate [19]. The concentration of these VFAs was found to have an impact on both cell dry weight (CDW) and PHA productivity, owing to their interactive effects.

In this study, the impact of the proportion of mixed VFAs on cell dry weight (CDW) and PHA productivity was examined. Furthermore, cultivation conditions and various feeding strategies were studied to optimize and enhance PHA productivity. Moreover, the study investigated the mutual effect between the VFAs metabolism and the PHA biosynthesis of *Halomonas* sp. YLGW01 during culture for PHA production.

## Materials and methods

### Bacterial strains and growth media

*Halomonas* sp. YLGW01, which was isolated from the coast of South Korea and produced PHA from VFAs, was cultured for this study [22]. The cultured strains were prepared for long-term storage by treating them with 20% glycerol and then storing them in a deep freezer at − 80 °C. For flask-scale PHA production, a seed culture was prepared at a 5 mL scale in marine broth (MB, Difco) by incubation at 30 °C for 24 h. The cultured cells were harvested by centrifugation at 3500 rpm at 4 ℃ for 10 min, the supernatant was removed, and the cells were washed twice with sterile distilled water. For the mixture analysis, MB media with varying concentrations and compositions of VFAs were employed. To determine the preference of nitrogen sources, a minimum medium composed of synthetic seawater without a preexisting nitrogen source was supplemented with soytone, peptone, tryptone, yeast extract, and beef extract at a concentration of 5 g/L.

### Design of experiments and mixture analysis

To determine the optimal ratio of volatile fatty acids, acetate, lactate and propionate were used to produce PHA. A mixture analysis which was designed by the Minitab 18 program was performed in order to optimize the PHA production and cell growth for different ratios of the three VFAs. The experimental design by degree of lattice 2 included 10 combinations of VFAs ratios, with VFAs ratios of 0.00, 0.17, 0.33, 0.5, 0.67, and 1.00. The concentrations of the mixed VFAs in each flask were determined to be lactate 0–3 g/L, acetate 0–20 g/L and propionate 0–9 g/L according to a previous study [13]. Each experimental sample was cultured at 30 °C and 200 rpm for 48 h in duplicate. In the data analysis, coefficients with p-values less than 0.1 were used.

### Fed-batch fermentation

The fed-batch was conducted in a 10 L fermentor (CNS, Korea) with a working volume of 5 L. The inoculum was prepared in 500 mL of MB medium and incubated at 200 rpm and 30 °C for 24 h. The production medium used was a synthetic sea water medium containing 9 g/L of acetate and 1.65 g/L of lactate as initial carbon sources. The main culture was carried out at pH 7, 30 °C, and 600 rpm for 90 h. In the DO-stat fed-batch, a feeding solution containing 9 g/L of acetate and 1.65 g/L of lactate was concentrated 20-fold and used. Additionally, a 20% soytone solution was used as a nitrogen source for feeding. The VFAs consumption-based fed-batch strategy was also employed, similar to the DO-stat fed-batch, where the feeding solution was adjusted proportionally to the VFAs consumption rate. In the case of the pH stat fed-batch, VFAs were utilized as a pH acid buffer in the same ratio, and only the 20% soytone solution was used for feeding. The soytone feeding solution for the pH stat fed-batch was added in real-time based on absorbance measured at 600 nm, and it was added when the OD value did not increase compared to the previous time points (26 h, 46 h, 56 h).

### Analytical methods

Analysis of the PHA contents in cells was performed by gas chromatography (GC) techniques and methodologies used in previous studies [23]. The fermentation broth was centrifuged at 3500 rpm for 10 min and washed with distilled water. The washed cells were transferred to a glass vial and then dried with a lyophilizer for 24 h to determine the cell dry weight (CDW). Chloroform and 15% (v/v) H_2_SO_4_/85% methanol were added with the same volume (1 mL each) and methanolysis was conducted for 2 h at 100 °C. After methanolysis, 1 mL of distilled water was added to the methyl ester solution and vortexed for 5 s. The chloroform layer was drawn off the glass vial into a microtube containing crystalline anhydrous Na_2_SO_4_ to remove the residual water. Filtered 1 μL aliquots of samples were injected into a gas chromatograph with a split mode (1/10) (Young-lin 6500, Seoul, Korea) equipped with a fused silica capillary column (Agilent HP-FFAP, 30 m × 0.32 mm, i.d. 0.25 μm film) and flame ionization detector (FID). The inlet temperature was 210 °C, and helium was supplied as a carrier gas at 3 mL/min. The oven temperature was controlled by following a gradient program of 0–5 min at 80 °C and then 12–17 min at 220 °C. The FID temperature was maintained at 230 °C throughout the operation. The concentrations of the carbon sources containing VFAs were determined by high-performance liquid chromatography (Agilent Technologies, USA) using a refractive index detector (RID) and an Aminex HPX-87H ion exclusion column (BioRad, USA). The mobile phase was 5 mM H_2_SO_4_, and the flow rate was 0.6 mL/min with the column temperature maintained at 60 °C. Cell growth was monitored by measuring the optical density (OD) using a spectrophotometer (Thermo Spectronics, Genesys 20, USA).

## Results and discussion

### Optimization of VFAs ratio for PHA production based on the mixture analysis

**Fig. 1:**
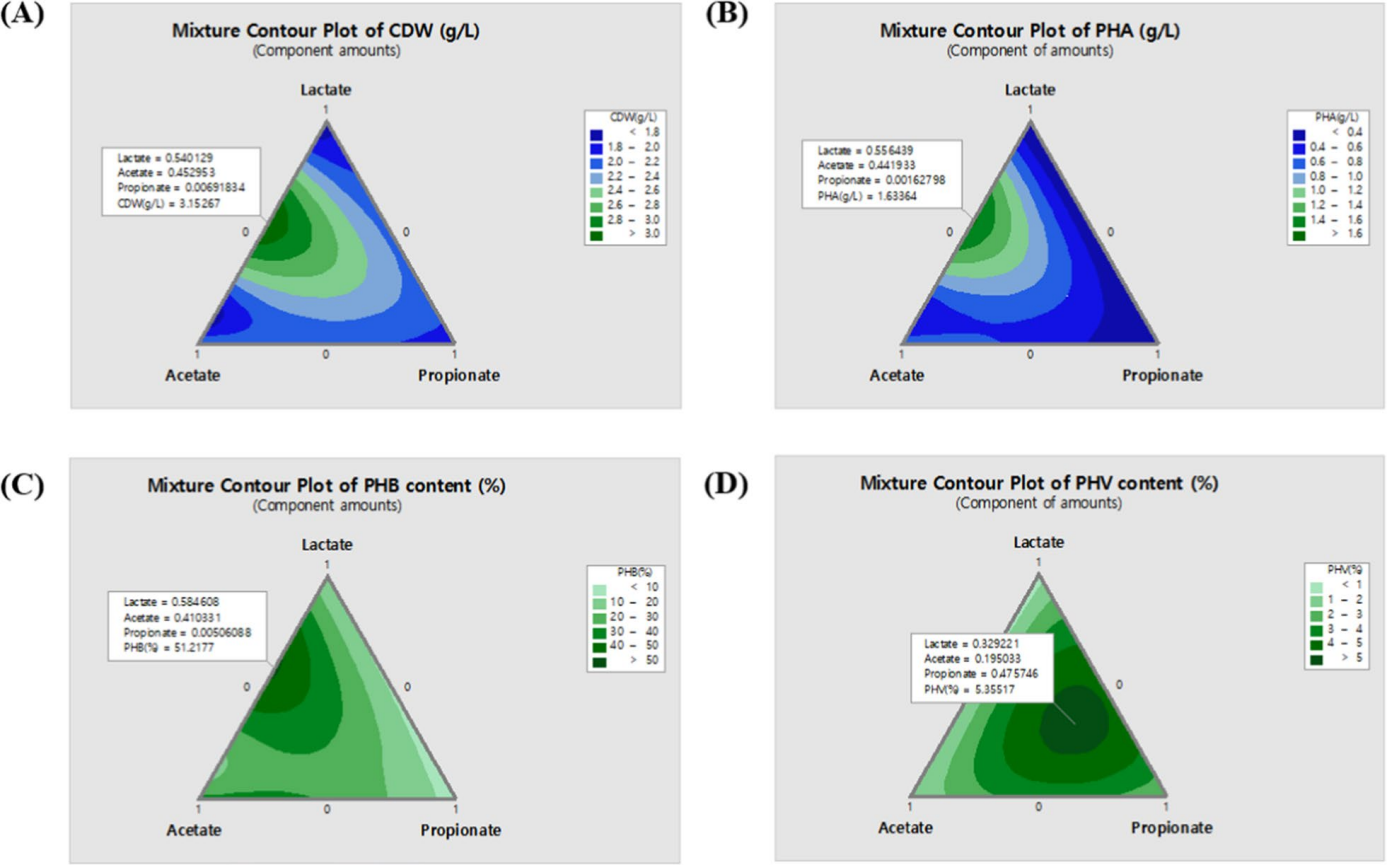
Contour plots for optimization of VFAs composition. **A** CDW (g/L), **B**: PHA titer (g/L), **C**: PHB content (%) and **D**: PHV (g/L) of different VFAs compositions. Based on the produced amount of PHA, the optimal composition ratio between lactate, acetate, propionate was determined as 0.55:0.45:0 for further study

In our previous study, it was observed that the utilization of VFAs, including acetate, lactate, and propionate, present in food wastewater, along with the optimization of their mixing ratios, led to an increase in PHA production. However, the growth inhibition concentrations of the strains varied significantly depending on the concentration of each volatile fatty acid. For acetate, the resistance concentration was 20 g/L, for lactate, it was 3 g/L, and for propionate, it was 9 g/L [13]. Therefore, when using mixed VFAs extracted from actual biomass, it is more efficient to adjust the overall concentration of the VFAs while maintaining the original mixing ratio rather than adjusting the concentration of each individual VFA used as a carbon source. In this study, we investigated the effects of different concentration ratios of the three VFAs on strain growth, PHA production, and the molar ratio of PHV via a mixture analysis of the three VFAs. The VFAs ratios and the corresponding experimental data are shown in Table 1, and based on the experimental data, contour plots were generated to predict the optimized data for each ratio (Fig. 1).

**Table 1:** Experimental design points for the mixture analysis model and their experimental results

No	Lactate	Acetate	Propionate	CDW (g/L)	PHA (g/L)	PHB content (%)	PHV content (%)
1	1	0	0	1.725	0.178	9.696	0.512
2	0.5	0.5	0	3.185	1.579	48.732	0.798
3	0	1	0	2.365	0.8	33.127	0.561
4	0	0.5	0.5	1.95	0.632	29.642	3.204
5	0	0	1	1.99	0.112	5.5	2.221
6	0.5	0	0.5	2.18	0.332	10.442	5.06
7	0.3333	0.3333	0.3333	2.48	0.943	32.815	4.909
8	0.6667	0.1667	0.1667	2.395	0.812	30.754	0.812
9	0.1667	0.1667	0.6667	2.21	0.426	15.227	4.052
10	0.1667	0.6667	0.1667	2.135	0.656	26.684	4.035

The results showed that strain growth is predicted to be highest with a ratio of 0.54 for lactate and 0.45 for acetate, resulting in a CDW value of 3.15 g/L (Fig. 1A). On the other hand, PHA titer is predicted to be maximized at a ratio of 0.55 for lactate and 0.44 for acetate, with a value of 1.63 g/L (Fig. 1B), and the PHA content is predicted to be 51.2% when lactate is present at a ratio of 0.58 and acetate at a ratio of 0.41 (Fig. 1C). Meanwhile, the molar fraction of 3HV is predicted to be 5.4% when lactate is present at a ratio of 0.33, acetate at 0.2, and propionate at 0.48 (Fig. 1D). This suggests that the molar fraction of 3HV is influenced by the concentration of propionate and related metabolic pathways.

When comparing these results with those of a previous study [13], it can be inferred that *Halomonas* sp. YLGW01 utilizes acetate as the main carbon source for cell growth. Furthermore, comparing the quantity and content of the produced PHB between the two studies, it was observed that under the response surface methodology based on the optimized concentrations of the previous study, the maximum conversion rate of PHB per gram of VFAs was 0.097 g for acetate and 0.31 g for lactate. In contrast, in the present study, the respective PHA conversion rates per gram of VFAs were 0.18 g for acetate and 0.95 g for lactate, indicating that the conversion rates of PHA are influenced not only by the concentration of the mixed VFAs but also by the mixing ratio.

### Examining the impact of additional nutrient sources in PHA production

**Fig. 2:**
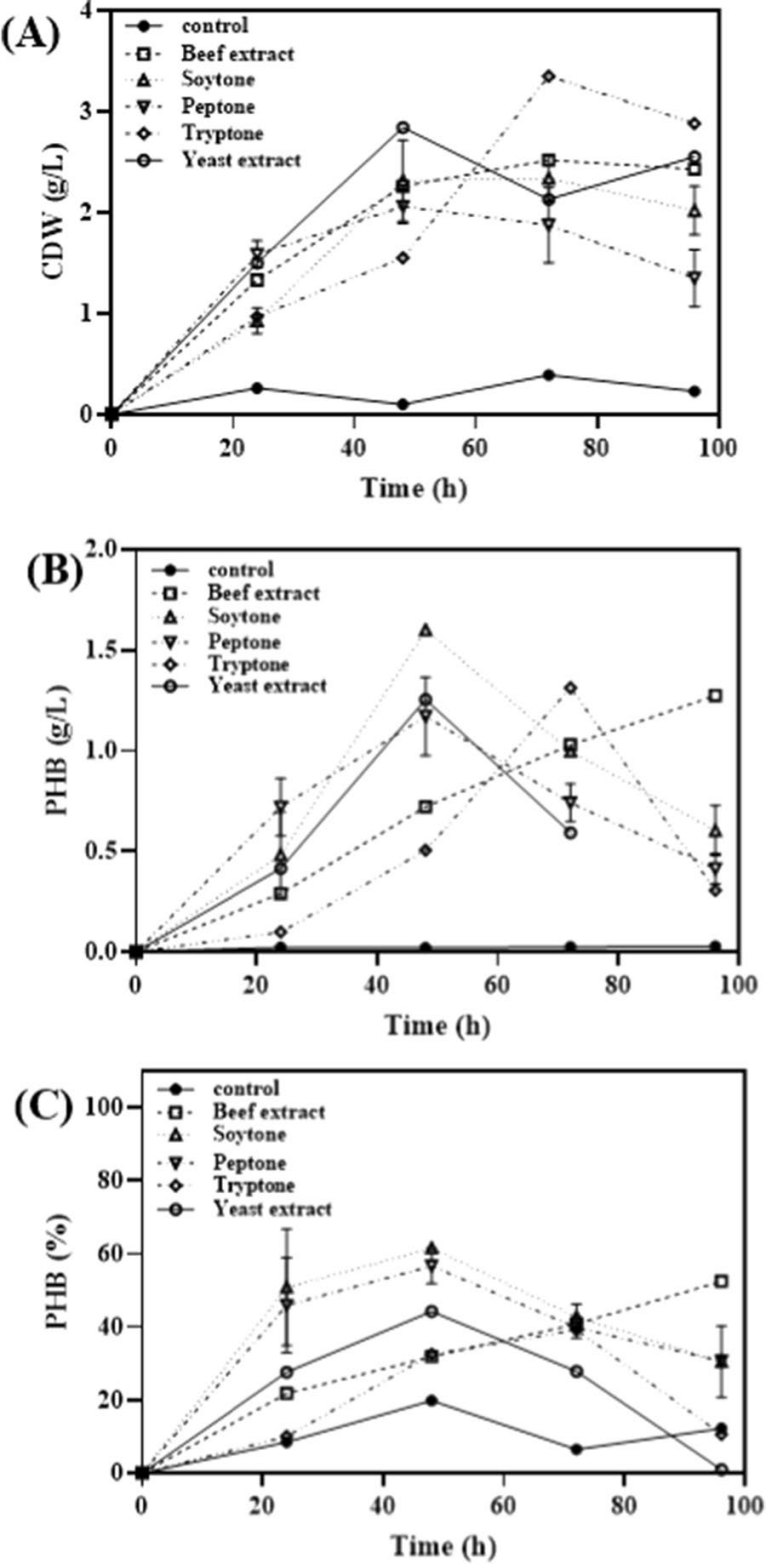
Effects of different complex nitrogen sources on PHA production. Each complex nitrogen sources were used as 5/gL for PHB production. **A**: CDW (g/L), **B**: PHB titer (g/L), **C**: PHB content (%)

**Fig. 3:**
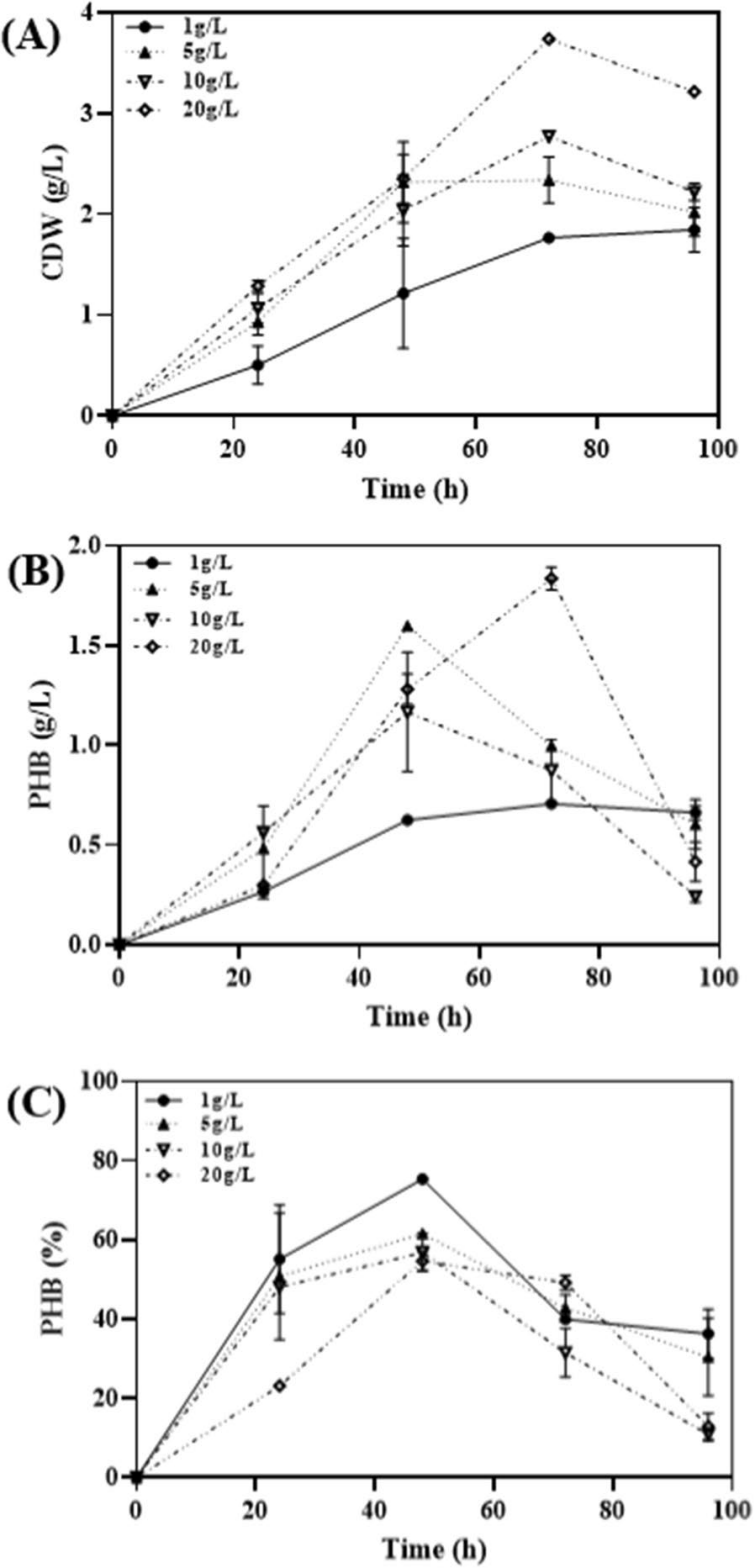
Effect of soytone concentration on PHA production. Soytone concentration was used in range of 1 to 20 g/L to examine its effect on PHA production. **A**: CDW (g/L), **B**: PHB titer (g/L), **C**: PHB content (%)

Nitrogen is a critical factor in PHA production as PHA synthesis typically occurs under conditions of excess carbon, but limited nitrogen or phosphorus availability [24]. In order to optimize PHA productivity based on changes in the C/N ratio, NH_4_Cl, an inorganic nitrogen source, was used to investigate PHA production at different C/N ratios (1 to 80). The results showed that the highest PHA production was observed at a C/N ratio of 40 (Table 2). Additionally, the impact of organic nitrogen sources on cell dry weight (CDW) and PHA production was examined by adding beef extract, soytone, peptone, tryptone, and yeast extract at a concentration of 5 g/L (Fig. 2). It was found that CDW was highest at 72 h with tryptone at 3.35 g/L (Fig. 2A), while PHB production and PHB content were 1.3 g/L and 39.2%, respectively. In contrast, soytone exhibited the highest PHB production and PHB content at 1.6 g/L and 61.6%, respectively, even though CDW at 48 h with soytone was not particularly higher than the other nitrogen sources (Fig. 2B, C). Subsequently, the changes in CDW and PHA production were monitored with varying the concentration of soytone. It was observed that CDW increased with higher concentrations of the nitrogen source (Fig. 3A). PHB production, on the other hand, reached its highest level at 1.84 g/L at 72 h with a soytone concentration of 20 g/L. However, at 48 h with a soytone concentration of 5 g/L, PHB production was 1.6 g/L (Fig. 3B). This indicates that the production efficiency of PHB per gram of soytone was approximately 0.09 g for 20 g/L, while it was approximately 0.32 g for 5 g/L, showing a production efficiency that was about 3.6 times higher. Therefore, considering the production efficiency of PHB relative to the concentration of soytone, it can be concluded that the optimal condition for PHB production is at a concentration of 5 g/L, which demonstrated a higher PHB production efficiency compared to the concentration of 20 g/L, which yielded the highest PHB production. Therefore, soytone can be considered the optimal nitrogen source for PHB production. One of the reasons why *Halomonas* sp. YGLGW01 shows better growth and PHA production in organic nitrogen sources than inorganic nitrogen sources may be attributed to the presence of various amino acids and derivatives in organic nitrogen sources, which are absent in inorganic nitrogen sources.

**Table 2:** Effect of C/N ratio variance on the cell growth and PHA production of *Halomonas* YLGW01

C/N ratio	CDW (g/L)	PHB (g/L)	PHB content (%)
1	0.2	0.048	24
10	0.23	0.069	30
20	0.28	0.0.67	23.93
40	0.3	0.1	33.33
80	0.33	0.087	26.36
∞	0.3	0.074	24.67

### Evaluation of PHA production by DO stat fed-batch strategy

To enhance PHA synthesis from PHA-producing strains and maximize productivity, different fed-batch strategies can be employed, taking into account the strain's metabolic characteristics, key carbon sources, and other crucial factors relevant to PHA production. Among them, feeding methods based on DO stat, pH stat, or substrate consumption rate have been recognized as effective approaches to significantly enhance PHA productivity [20, 25, 26]. For instance, using *C. necator* H16 strain, a pH stat-based feeding approach with mixed VFAs resulted in a biomass concentration of 112.4 g/L and 83% PHB production [20]. In the case of *Halomonas*, several studies on PHA production through fed-batch using glucose have been conducted [27–29]. In one study, acetate was used as a sole carbon source, resulting in a biomass concentration of 70 g/L and PHB production of 50 g/L by manual fed-batch culture [30]. In our previous study involving mixed VFAs, including acetate, lactate, butyrate, and propionate, a manual fed-batch culture achieved a biomass concentration of 12.3 g/L and PHA production of 6.6 g/L [13]. Most of the fed-batch strategies conducted so far have focused on manually maintaining the concentration of carbon sources. Research on feed control based on DO stat or pH stat has not been performed. Therefore, in this study, experiments were conducted to investigate how PHA productivity varies with different feeding strategies in *Halomonas* and to determine the optimal conditions for PHA production.

**Fig. 4:**
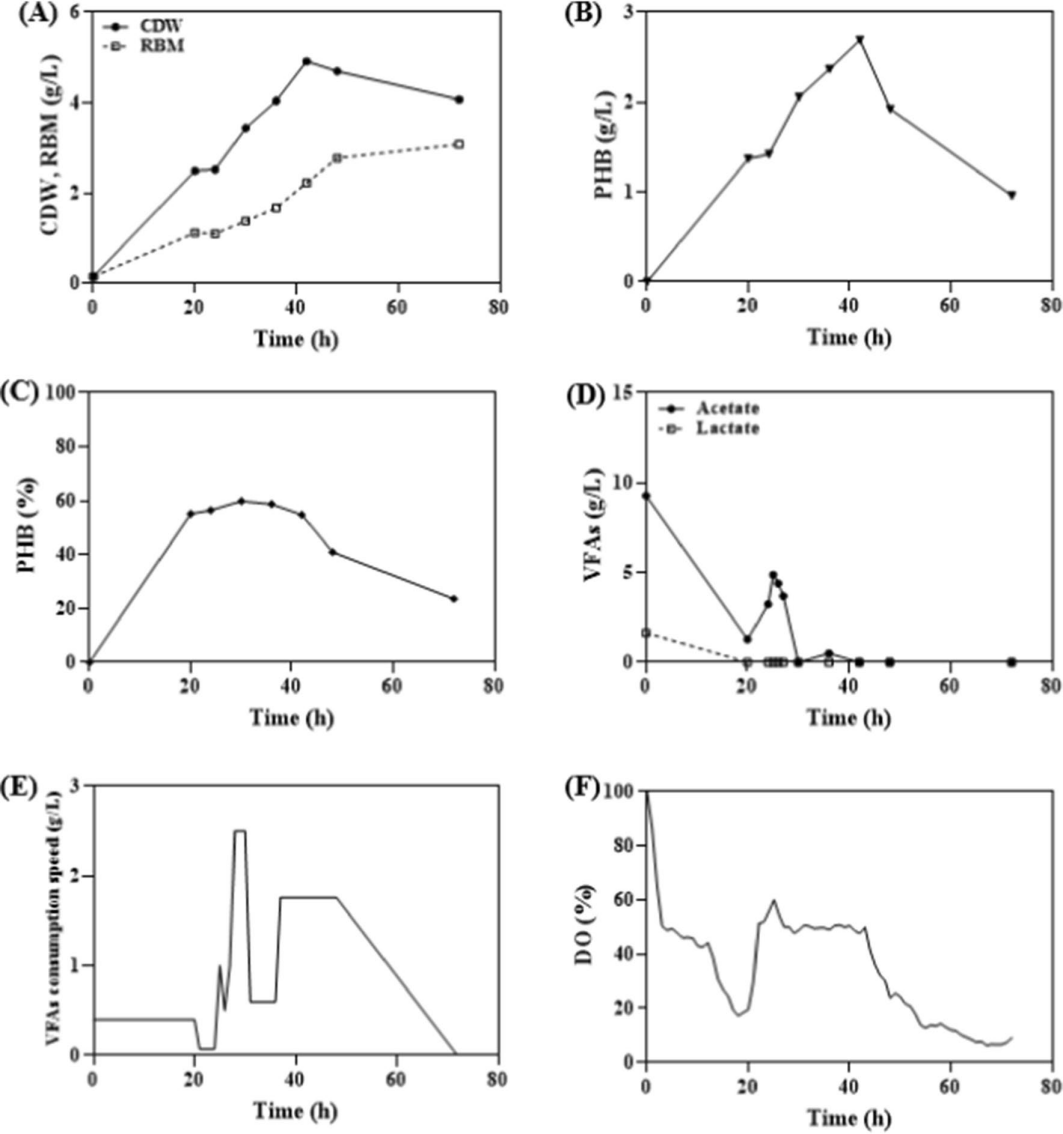
DO-stat fed batch culture for PHA production. Depletion of VFAs was occurred within 48 h. **A**: CDW, Residual biomass (g/L) **B**: PHB titer (g/L), **C**: PHB content (%), **D**: VFAs concentration (g/L), **E**: VFAs consumption speed (g/L), **F**: DO (%)

Firstly, for the DO stat-based feed control method, a feeding solution consisting of soytone (total concentration of 5 g/L) and acetate:lactate in a ratio of 0:45:0.55 (acetate 9 g/L, lactate 1.65 g/L) was used. The feeding conditions were set based on previous studies [13], with cultivation initiated when the DO reached above 50% at the point where it decreased and then increased (Fig. 4). As a result, within 42 h, the CDW reached 4.92 g/L, and PHB production was observed to be up to 2.69 g/L (Fig. 4A and B). Additionally, the PHB content reached around 56% within 24 h and showed a tendency to be maintained until 42 h (Fig. 4C). Furthermore, the VFAs concentrations in the culture showed that acetate reached a concentration of 1.28 g/L within 20 h after the initiation of cultivation, while lactate was completely consumed. Afterward, the acetate concentration increased up to 3.24 g/L within 24 h and then exhibited a decreasing trend (Fig. 4D). These observations can be attributed to the phenomenon where the DO value decreased by approximately 20% around 20 h, sharply increased to above 50% after 24 h, remained stable until 42 h, and then steadily decreased over time (Fig. 4E). Based on the calculation of the VFAs consumption rate for each interval, it was observed that the consumption rate remained relatively stable until almost complete consumption of the VFAs within 22 h. However, after that point, as the DO remained consistently high for a certain period, the VFAs consumption rate increased significantly up to 2.5 g/L/h, indicating a rapid influx of VFAs in response to the DO-based feeding condition. Subsequently, the consumption rate maintained a relatively stable level in accordance with the feeding conditions dictated by the DO. As the DO gradually decreased, both the feeding and consumption rates declined, eventually leading to the cessation of feeding and a decrease in the consumption rate (Fig. 4F). This can be attributed to the continuous feeding of VFAs, which leads to an increase in cell mass and a significant decrease in dissolved oxygen levels. Unlike *C. necator*, where PHA productivity is directly influenced by DO concentration, both PHA production and cell growth seems to occur simultaneously in *Halomonas* sp. YLGW01. As a result, the DO level cannot be maintained and continuously decreases. It is generally known that the growth phase and PHA storage mechanism in microorganisms act in an inverse relationship to each other [31, 32]. However, considering the continuous increase in residual cell mass of *Halomonas* sp. YLGW01 due to the continuous feeding of VFAs until 42 h, it is speculated that the growth phase and PHA storage mechanism do not act in an inverse relationship when utilizing VFAs. Therefore, in order to maintain high PHA production by DO stat-based feeding, it is necessary to set variable DO values based on the results obtained through repeated manual experiments.

### Evaluation of PHA production by VFAs consumption rate stat fed-batch strategy

**Fig. 5:**
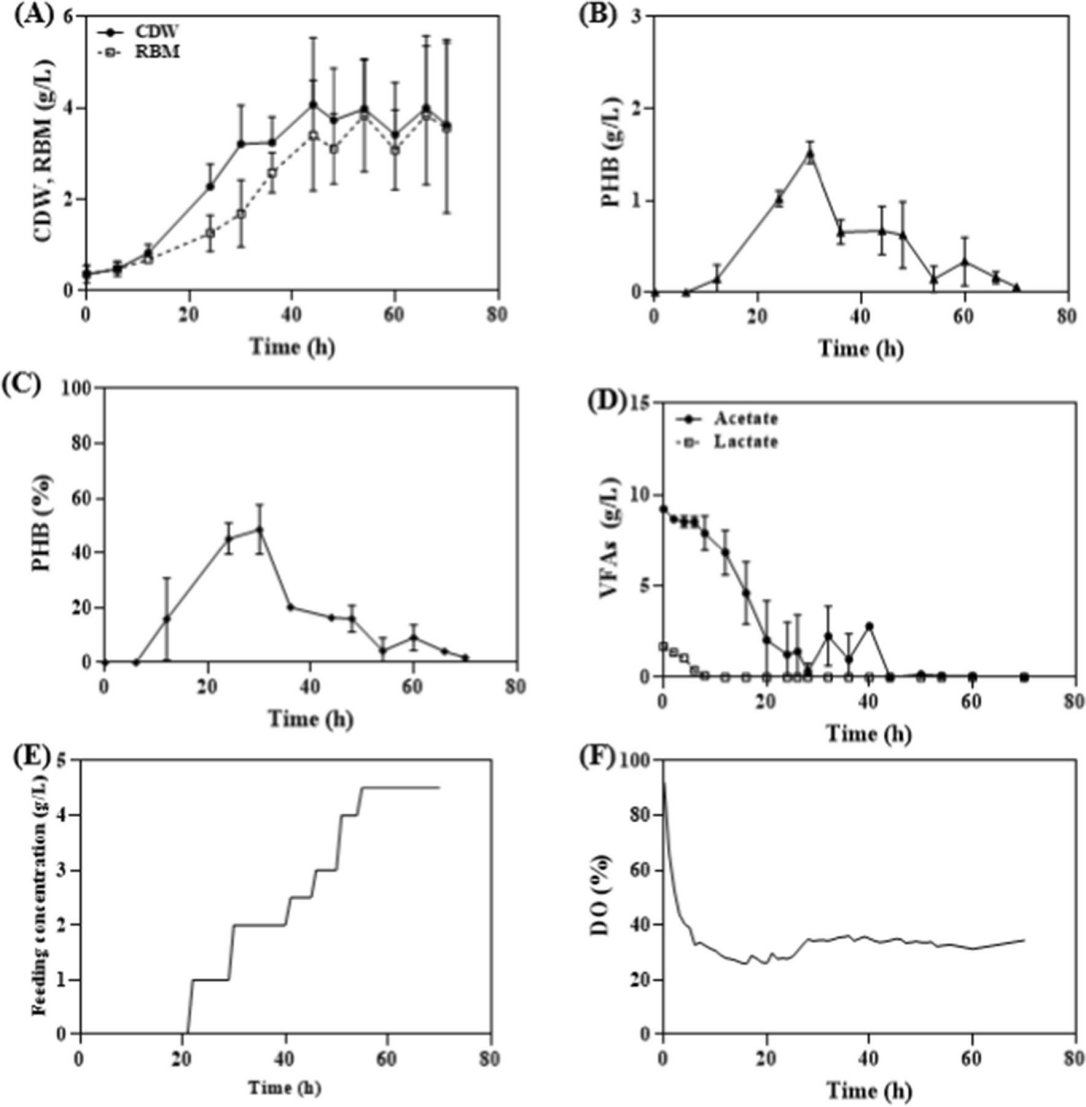
VFAs consumption rate stat fed batch for PHA production. Depletion of VFAs was occurred within 28 h and 44 h during the cultivation. Decreased produced PHB amount was observed from 28 h. **A**: CDW, Residual biomass (g/L) **B**: PHB titer (g/L), **C**: PHB content (%), **D**: Organic acid concentration (g/L), **E**: Feeding concentration (g/L), **F**: DO (%)

To resolve the rapidly increasing required feeding amount of VFAs during culture, and maintain the VFAs concentration based on the culture, feed control was carried out based on the average consumption rate of VFAs per unit dry weight of *Halomonas* sp. YLGW01 (Fig. 5). The results showed that CDW and PHA production increased to 3.21 g/L and 1.53 g/L, respectively, at 30 h. However, the amount of produced PHA was dramatically reduced thereafter (Fig. 5AB). This difference was more pronounced in the change of PHB content, which increased up to 49% at 30 h but sharply decreased to below 10% thereafter (Fig. 5C). The VFAs concentration also showed a continuous decrease until 30 h, followed by maintaining an approximate 2.3 g/L concentration until 44 h before decreasing again. This can be attributed to the continuous increase in feeding concentration based on the dry weight, starting from 24 h, up to a maximum of 4.5 g/L/h, reaching the volume limit of the culture medium (Fig. 5DE). Despite this effort, the VFAs consumption rate increased significantly over time. This is the likely reason for the inability to maintain PHA content, resulting in its decrease. Furthermore, the reason for the sharp increase in the VFAs consumption rate and the decrease in PHA content can be attributed to the rapid increase in the consumption rate and the decrease in the concentration of the VFAs in the culture medium. In contrast, when maintaining the VFAs concentration through feed control, the DO value remained relatively stable without significant changes, as compared to the DO-stat fed-batch conditions (Fig. 5F). Therefore, it can be concluded that DO stat or VFAs consumption rate stat-based feed control is not suitable for *Halomonas* sp. YLGW01 when using VFAs as a carbon source during fed-batch production of PHA. Unlike *C. necator*, *Pseudomonas*, or engineered *E. coli*, *Halomonas* sp. YLGW01 are highly sensitive to VFAs concentrations, and their PHA synthesis or degradation metabolism appears to respond closely to changes in the VFAs levels. Additionally, when producing PHA using hexose-based carbon sources, even when the carbon source is nearly exhausted, the PHA content remains stable for a certain period of time [33]. This suggests a closer association between VFAs metabolism and PHA synthesis in *Halomonas* sp. YLGW01 compared to other microbial strains.

### Effect of soytone feeding for PHA production based on the pH stat fed-batch strategy

**Fig. 6:**
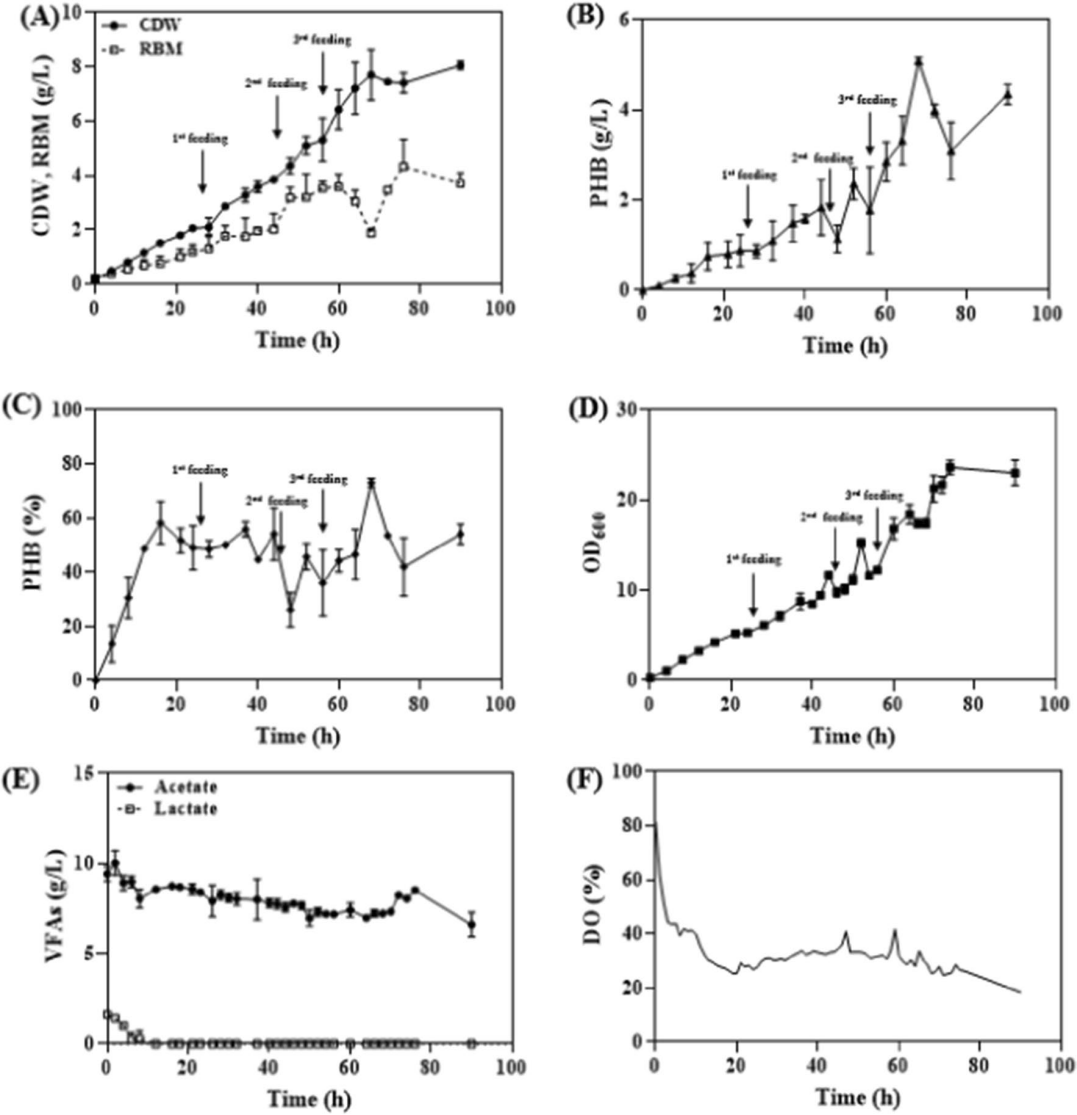
pH stat soytone fed-batch culture for PHA production. 20% of soytone was fed in three times during the cultivation on the basis that there was no change in OD value. **A**: CDW, Residual biomass (g/L), **B**: PHB titer (g/L), **C**: PHB content (%), **D**: OD_600_, **E**: Organic acid concentration (g/L), **F**: DO (%)

In order to increase PHA productivity and maintain the produced PHA content in *Halomonas* sp. YLGW01, feed control should be carried out with a constant VFAs concentration, and a supply of inorganic or organic nitrogen sources is necessary for strain growth. Additionally, it was observed that the pH increases as VFAs are consumed by *Halomonas* sp. YLGW01 (data not shown). Considering the pH increase when utilizing VFAs as carbon sources and the low pH of VFAs, pH–stat-based VFAs feed control was performed in *Halomonas* sp. YLGW01. Furthermore, sequential feeding was conducted with a total concentration of 5 g/L of soytone, monitoring the OD values and adding soytone when growth no longer increased (Fig. 6). As a result, the OD of *Halomonas* sp. YLGW01 reached a plateau at 26, 46, and 56 h, and the addition of soytone led to further increases in CDW (Fig. 6a). The maximum growth reached 8.1 g/L within 90 h, and residual biomass(RBM) showed a gradual increase. The amount of produced PHA also increased after soytone feeding but then decreased after a certain period before recovering, reaching a maximum of 5.1 g/L at 68 h (Fig. 6B). A similar trend can be observed in the change of PHA content (%), where the content increases with each soytone feeding, reaching a maximum of 73% at 68 h (Fig. 6C). Subsequently, as no further soytone supplementation was provided, the content decreased to 42% at 76 h and then increased again to 54% at 90 h. Considering the minimal difference in RBM between DO-stat and VFAs consumption rate-stat fermentation, it can be inferred that with a constant VFAs concentration, the produced PHA content is maintained at a relatively stable level. Particularly, when feeding based on the pH-dependent acetate and lactate ratio, it is speculated that *Halomonas* sp. YLGW01 prefers lactate over acetate as a carbon source (Fig. 6D). Furthermore, the VFAs consumption rate generally increases with the increase in CDW, but at the point where soytone is considered fully consumed, the VFAs consumption rate dramatically decreases before recovering (Fig. 6E). Regarding DO, it remains relatively stable, similar to the VFAs consumption rate-stat (Fig. 6F).

Based on these findings, for effective fed-batch production using *Halomonas* sp. YLGW01 with mixed VFAs, it is recommended to implement feeding control that responds sensitively to the VFAs concentration, considering the characteristic response of *Halomonas* sp. YLGW01. Maintaining a constant acetate concentration based on pH–stat control proves to be the most effective approach. Additionally, the results demonstrate that soytone feeding is beneficial for the growth of *Halomonas* sp. YLGW01 compared to NH_4_Cl alone. Therefore, to further enhance PHA production from *Halomonas* sp. YLGW01 using VFAs, optimization of soytone or inorganic nitrogen source feeding is necessary. Furthermore, strain development through the introduction of CoA transferase related to VFAs conversion or the deletion of depolymerase genes could be considered.

## Conclusion

In this study, it was demonstrate that *Halomonas* sp. YLGW01 utilizes mixed VFAs as carbon sources with an acetate-to-lactate ratio of 0.45:0.55 resulting in the highest cell dry weight (CDW) of 3.15 g/L and PHA production of 1.6 g/L. Furthermore, it was found that supplementation with organic nitrogen sources, such as soytone, instead of inorganic nitrogen sources can increase PHA production. On the other hand, it observed that *Halomonas* sp. YLGW01 exhibit a sensitive response to the external VFAs concentration when utilized as a carbon source, leading to a rapid fluctuation in PHA content. To address this issue, it applied various feeding strategies, and it was demonstrated that pH stat-based control of VFAs feeding can effectively enhance PHA productivity as pH increases due to strain growth and PHA production. While the PHA productivity still needs improvement, these results can serve as valuable data for optimizing large-scale PHA production using VFAs by *Halomonas* sp. YLGW01 in the future.

## Data Availability

The data that support the findings of this study are available on request from the corresponding author. The data are not publicly available due to privacy or ethical restrictions.
